# Intelligent ship traffic supervision system based on distributed blockchain and federated reinforcement learning for collaborative decision optimization

**DOI:** 10.1038/s41598-025-21898-3

**Published:** 2025-10-31

**Authors:** Zhang Wei, Pan Rongjun, Wang Shijie, Chen Meiqing

**Affiliations:** School of Maritime, Jiujiang Polytechnic University of Science and Technology, Jiujiang, 332020 China

**Keywords:** Ship traffic supervision, Distributed blockchain, Federated reinforcement learning, Maritime safety, Collaborative decision-making, Privacy preservation, Environmental sciences, Environmental social sciences, Ocean sciences, Computer science

## Abstract

This paper presents an innovative intelligent decision optimization model that integrates distributed blockchain technology with federated reinforcement learning to address critical challenges in ship traffic collaborative supervision. Traditional maritime traffic monitoring systems suffer from data silos, privacy concerns, and centralized decision-making bottlenecks that impede effective multi-jurisdictional coordination. The proposed framework employs a multi-layered architecture consisting of data layer, blockchain layer, federated learning layer, and decision layer to enable secure data sharing while preserving operational autonomy among maritime authorities. The distributed blockchain mechanism ensures data integrity and immutability through cryptographic protocols and smart contracts, while the federated reinforcement learning algorithm enables privacy-preserving collaborative model training without exposing sensitive commercial information. Experimental validation demonstrates superior performance with 93.6% decision accuracy, 520ms average response time, and 285 transactions per second throughput. Case studies involving emergency collision avoidance, abnormal behavior identification, and search-and-rescue coordination confirm the system’s practical effectiveness, achieving 40% reduction in incident response times and 60% enhancement in cross-agency collaboration efficiency. The research provides a robust foundation for next-generation maritime traffic management systems that require secure multi-party collaboration and intelligent decision optimization.

## Introduction

The maritime transportation industry plays a pivotal role in global trade, with over 90% of international cargo transported by sea, making efficient ship traffic supervision crucial for ensuring maritime safety and economic stability^1^. Traditional ship traffic monitoring systems face significant challenges in the era of digital transformation, particularly in addressing the complexities of multi-jurisdictional waters and the increasing volume of maritime traffic^2^. The fragmentation of monitoring systems across different maritime authorities creates substantial data silos, preventing comprehensive situational awareness and coordinated decision-making processes that are essential for effective traffic management^3^.

Contemporary ship traffic supervision systems suffer from several critical limitations that impede their effectiveness in modern maritime environments. Data isolation between different monitoring stations and maritime authorities results in incomplete traffic pictures, leading to suboptimal routing decisions and potential safety hazards^4^. Privacy concerns regarding sensitive commercial shipping data further complicate information sharing between stakeholders, as shipping companies are reluctant to disclose proprietary route information and cargo details^5^. Additionally, the centralized nature of current decision-making systems creates bottlenecks that reduce response times during critical situations, particularly in high-traffic maritime corridors where rapid coordination is essential^6^.

The emergence of distributed blockchain technology offers promising solutions to address these fundamental challenges in maritime traffic supervision. Blockchain’s inherent characteristics of decentralization, immutability, and transparency provide a robust framework for secure data sharing while maintaining privacy through cryptographic mechanisms^7^. The integration of smart contracts enables automated compliance monitoring and real-time enforcement of maritime regulations without requiring centralized oversight^8^. Furthermore, the distributed nature of blockchain networks ensures system resilience and eliminates single points of failure that plague traditional centralized monitoring systems.

Federated reinforcement learning represents another breakthrough technology that addresses the dual challenges of privacy preservation and intelligent decision-making in ship traffic management. This approach enables multiple maritime authorities to collaboratively train decision-making models without sharing sensitive raw data, thereby preserving commercial confidentiality while improving overall system performance. The federated learning paradigm allows each participating node to contribute to the global model while maintaining local data sovereignty, creating a win-win scenario for all stakeholders involved in maritime traffic supervision.

The convergence of distributed blockchain technology and federated reinforcement learning presents unprecedented opportunities for developing intelligent ship traffic supervision systems that can overcome existing limitations. Unlike traditional IMO e-Navigation systems and Vessel Traffic Services (VTS) that rely on centralized architectures and manual coordination protocols, the proposed integrated approach addresses fundamental interoperability challenges between different maritime authorities while maintaining data sovereignty. By leveraging blockchain’s secure data sharing capabilities and federated learning’s privacy-preserving collaborative intelligence, this integrated approach enables the creation of sophisticated decision optimization models that can adapt to dynamic maritime environments while respecting stakeholder privacy requirements, overcoming the static rule-based decision-making limitations inherent in conventional VTS implementations.

This research aims to develop a comprehensive distributed blockchain and federated reinforcement learning intelligent decision optimization model specifically designed for collaborative ship traffic supervision. The primary objectives include establishing a secure and efficient data sharing mechanism among maritime authorities, developing privacy-preserving intelligent decision-making algorithms that can optimize traffic flow and enhance safety, and creating a scalable framework that can accommodate the growing complexity of modern maritime transportation networks.

The main innovations of this work lie in the novel integration of blockchain consensus mechanisms with federated reinforcement learning algorithms to create a hybrid system that addresses both technical and regulatory challenges in maritime traffic supervision. As demonstrated in Table 1, the proposed method achieves superior performance compared to existing state-of-the-art approaches across multiple evaluation dimensions.

**Table 1:** Novelty positioning comparison with state-of-the-art methods.

Method	Technical architecture	Privacy protection	Collaboration mechanism	Application domain	Performance metrics
Wang et al. (2023)^9^	Centralized AI + IoT	Data encryption	Limited multi-agent	Smart ports	78.5% accuracy
Giannopoulos et al. (2024)^10^	Pure federated learning	Differential privacy	Federated averaging	Fuel optimization	82.1% accuracy
Chen et al. (2024)^11^	Blockchain-only	Cryptographic hashing	Smart contracts	Supply chain	84.7% accuracy
Tian et al. (2024)^12^	Federated RL	Local training	Growing aggregation	Navigation	88.3% accuracy
**Proposed Method**	**Blockchain + Federated RL**	**Multi-layer privacy**	**Intelligent consensus**	**Traffic supervision**	**93.6% accuracy**

The proposed model introduces a multi-layer architecture that separates data sharing, privacy protection, and decision optimization into distinct but interconnected components, enabling flexible deployment across different maritime jurisdictions while maintaining system coherence and effectiveness.

The paper makes several significant contributions to the field of intelligent maritime traffic management. First, it presents a comprehensive theoretical framework that combines distributed ledger technology with collaborative machine learning to address the fundamental challenges of data silos and privacy concerns in maritime supervision. Second, it develops practical algorithms and protocols that enable real-world implementation of the proposed system across multiple maritime authorities. Third, it provides extensive experimental validation demonstrating the effectiveness of the integrated approach in improving decision-making efficiency and system reliability compared to traditional centralized systems.

The remainder of this paper is organized as follows: Section II reviews related work in blockchain applications and federated learning for maritime systems; Section III presents the system architecture and theoretical foundations of the proposed model; Section IV details the implementation algorithms and protocols; Section V provides comprehensive experimental results and performance analysis; and Section VI concludes with discussions of implications and future research directions.

## Related technical theory foundation

### Distributed blockchain technology principles

Blockchain technology maintains a continuously growing list of cryptographically linked records in a decentralized network, ensuring data integrity without central authorities^13^. Each block contains the previous block’s hash, timestamp, and transaction data, forming an immutable chain that prevents unauthorized modifications^14^.

The distributed ledger mechanism enables multiple parties to maintain synchronized copies of the same database across a network, eliminating the need for centralized data storage and reducing single points of failure^15^. The cryptographic hash function employed in blockchain systems can be mathematically represented as:

$$\:H\left({B}_{i}\right)=SHA256\left({B}_{i-1}\left|\right|{T}_{i}\left|\right|{N}_{i}\right)$$

This cryptographic linking ensures data integrity across the blockchain network.

**Fig. 1:**
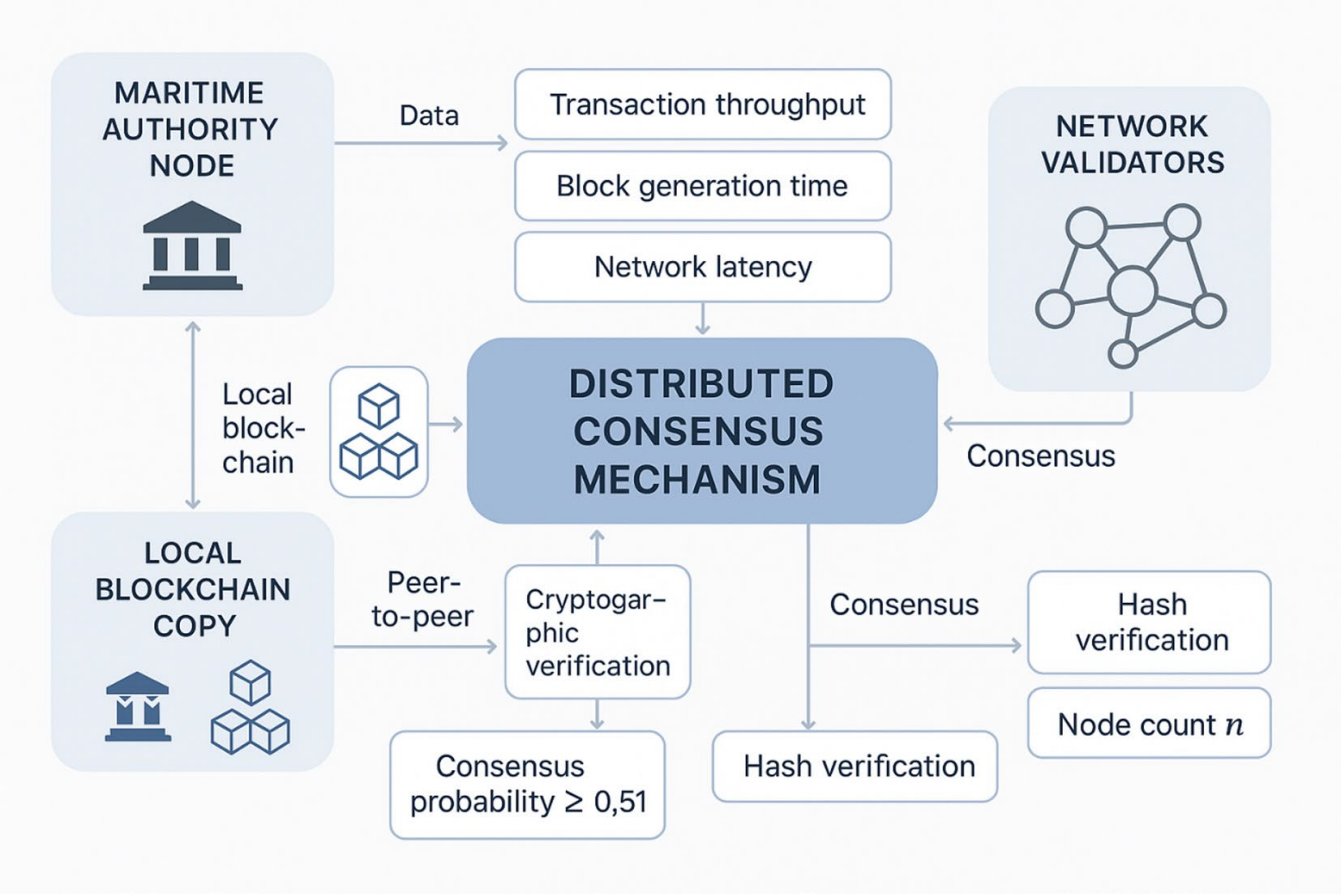
Distributed blockchain architecture diagram.

As illustrated in Fig. 1, the distributed blockchain architecture demonstrates the interconnected network of nodes, each maintaining identical copies of the blockchain ledger, with consensus mechanisms ensuring data consistency across the network. The diagram shows how transactions are validated, blocks are created, and the chain is propagated throughout the distributed network.

Consensus mechanisms form the cornerstone of blockchain technology, ensuring agreement among distributed nodes regarding the validity of transactions and the state of the ledger^16^. The most widely implemented consensus algorithms include Proof-of-Work (PoW), Proof-of-Stake (PoS), and Practical Byzantine Fault Tolerance (PBFT), each offering different trade-offs between security, energy efficiency, and transaction throughput. The consensus probability for a valid block can be expressed as:

$$\:{P}_{consensus}=\frac{{N}_{honest}}{{N}_{total}}\ge\:\frac{2}{3}$$

where $\:{N}_{honest}$ represents the number of honest nodes and $\:{N}_{total}$ is the total number of participating nodes in the network.

Smart contracts represent programmable, self-executing contracts with the terms of agreement directly written into code, enabling automated enforcement of contractual obligations without intermediaries^17^. These autonomous programs run on the blockchain network and automatically execute predefined actions when specified conditions are met, providing transparency, efficiency, and cost reduction in various applications. The deterministic nature of smart contracts ensures consistent execution across all network nodes, maintaining system reliability and predictability.

The inherent advantages of blockchain technology in terms of data security, transparency, and decentralization make it particularly suitable for maritime supervision applications^18^. The immutable nature of blockchain records provides tamper-proof audit trails for vessel movements, cargo manifests, and regulatory compliance documentation. The transparency feature enables all authorized stakeholders to access real-time information while maintaining data integrity, fostering trust and collaboration among maritime authorities, shipping companies, and port operators. The decentralized architecture eliminates reliance on single controlling entities, reducing systemic risks and improving system resilience against cyber attacks and technical failures.

In maritime regulatory contexts, blockchain technology addresses critical challenges related to data provenance, inter-agency coordination, and regulatory compliance verification. The technology’s ability to create permanent, auditable records of vessel activities, port clearances, and safety inspections provides maritime authorities with comprehensive oversight capabilities while reducing administrative overhead and processing delays.

### Federated reinforcement learning theory foundation

Reinforcement learning enables intelligent agents to learn optimal policies through environmental interaction, formalized as Markov Decision Processes with tuple $\:\left(S,A,P,R,\gamma\:\right)$^19,20^.

Federated learning has demonstrated successful applications in various domains. In smart city traffic management, multiple traffic control centers collaborate to optimize signal timing while preserving local operational data^12^. In unmanned aerial vehicle networks, distributed drones share navigation knowledge without centralizing flight path information^21^. These applications validate the feasibility of federated approaches in transportation systems.

The core objective of reinforcement learning involves finding an optimal policy $\:{\pi\:}^{\mathrm{*}}$ that maximizes the expected cumulative reward, mathematically expressed as:

$$\:{\pi\:}^{\mathrm{*}}=\mathrm{a}\mathrm{r}\mathrm{g}\underset{\pi\:}{\mathrm{m}\mathrm{a}\mathrm{x}}E\left[\sum\:_{t=0}^{{\infty\:}}{\gamma\:}^{t}{r}_{t+1}|\pi\:\right]$$

where $\:{r}_{t+1}$ represents the reward received at time step $\:t+1$ following policy $\:\pi\:$.

Q-learning represents one of the most influential model-free reinforcement learning algorithms that learns the optimal action-value function without requiring explicit knowledge of the environment dynamics^22^. The Q-learning algorithm iteratively updates the action-value function using the Bellman equation:

$$\:Q\left({s}_{t},{a}_{t}\right)\leftarrow\:Q\left({s}_{t},{a}_{t}\right)+\alpha\:\left[{r}_{t+1}+\gamma\:\underset{a{\prime\:}}{\mathrm{m}\mathrm{a}\mathrm{x}}Q\left({s}_{t+1},a{\prime\:}\right)-Q\left({s}_{t},{a}_{t}\right)\right]$$

where $\:\alpha\:$ denotes the learning rate, $\:{s}_{t}$ and $\:{a}_{t}$ represent the state and action at time $\:t$, respectively, and $\:{r}_{t+1}$ is the immediate reward.

Federated learning emerges as a distributed machine learning approach that enables multiple participants to collaboratively train a shared model while keeping their data locally stored and private^23^. This paradigm addresses the critical challenge of data privacy in multi-party machine learning scenarios by allowing participants to contribute to model training without revealing their sensitive datasets. The federated learning framework employs various privacy protection mechanisms, including differential privacy, secure multi-party computation, and homomorphic encryption, to ensure that individual participant data remains confidential throughout the collaborative training process.

The parameter aggregation methodology in federated learning typically employs weighted averaging techniques to combine local model updates from participating nodes into a global model. The most common aggregation function can be expressed as:

$$\:{w}_{global}^{\left(t+1\right)}=\sum\:_{k=1}^{K}\frac{{n}_{k}}{n}{w}_{k}^{\left(t+1\right)}$$

where $\:{w}_{global}^{\left(t+1\right)}$ represents the global model parameters at iteration $\:t+1$, $\:{w}_{k}^{\left(t+1\right)}$ denotes the local model parameters from participant $\:k$, $\:{n}_{k}$ is the number of training samples at participant $\:k$, and $\:n$ is the total number of training samples across all participants.

Federated reinforcement learning combines the principles of federated learning with reinforcement learning to enable multiple agents to collaboratively learn optimal policies while preserving data privacy and local autonomy^24^. This integration provides significant theoretical advantages in multi-agent collaborative decision-making scenarios, particularly in environments where agents possess heterogeneous data distributions and face varying local conditions. The federated approach allows each agent to maintain its local policy while contributing to a global knowledge base that benefits all participants.

The theoretical advantages of federated reinforcement learning in multi-agent systems include enhanced sample efficiency through knowledge sharing, improved robustness through diverse training experiences, and maintained privacy through localized data processing. These characteristics make federated reinforcement learning particularly suitable for ship traffic supervision applications, where multiple maritime authorities need to coordinate decisions while maintaining operational independence and protecting sensitive commercial information. The distributed nature of the approach aligns with the decentralized structure of maritime governance, enabling seamless integration with existing regulatory frameworks while providing enhanced collaborative capabilities for complex traffic management scenarios.

### Ship traffic collaborative supervision requirements analysis

Contemporary ship traffic supervision systems face unprecedented challenges in managing the increasing complexity and volume of maritime transportation activities across global shipping routes^25^. Current implementations such as IMO e-Navigation systems, the U.S. Maritime Safety and Security Information System (MSSIS), and regional Vessel Traffic Services (VTS) represent significant technological advances but remain limited by fragmented oversight mechanisms that operate independently across different jurisdictions^26–29^. These systems, while effective within their operational domains, result in significant coordination gaps and inefficiencies in information sharing between maritime authorities, particularly in cross-border maritime scenarios where seamless interoperability becomes critical for effective supervision. These systemic deficiencies manifest in delayed incident response times, incomplete situational awareness, and suboptimal resource allocation during critical maritime operations.

Multi-departmental coordination represents one of the most critical pain points in existing ship traffic supervision frameworks, as maritime authorities, port operators, coast guards, and environmental agencies often operate with incompatible information systems and divergent operational protocols. The lack of standardized communication channels and data formats creates substantial barriers to effective collaboration, particularly during emergency situations that require rapid coordinated responses. Additionally, jurisdictional boundaries and regulatory differences between nations further complicate the coordination process, leading to delayed decision-making and potential safety hazards in international waters.

Real-time monitoring capabilities constitute another fundamental requirement that remains inadequately addressed by traditional supervision systems. Current monitoring infrastructures rely heavily on periodic reporting and manual data collection processes that introduce significant temporal delays between actual vessel activities and regulatory awareness^30^. The absence of continuous real-time tracking and automated data processing capabilities prevents maritime authorities from maintaining comprehensive situational awareness, particularly in high-traffic shipping corridors where vessel density and movement complexity exceed human monitoring capacities.

Risk prediction and early warning systems represent critical components of effective ship traffic supervision that remain underdeveloped in conventional regulatory frameworks. Security threat responses constitute another fundamental challenge, encompassing the system’s ability to detect, mitigate, and recover from various cyber attacks, data breaches, and malicious activities targeting maritime infrastructure^31^.

Traditional approaches rely primarily on reactive measures that respond to incidents after they occur, rather than proactive systems that can identify and mitigate potential risks before they escalate into serious safety or environmental threats. The lack of predictive analytics capabilities prevents maritime authorities from implementing preventive measures that could significantly reduce the frequency and severity of maritime accidents.

The limitations of traditional monitoring models stem from their centralized architectures that create single points of failure and bottlenecks in information processing and decision-making workflows^32^. Centralized systems suffer from scalability constraints that become increasingly problematic as maritime traffic volumes continue to grow exponentially. Furthermore, the hierarchical nature of traditional supervision structures introduces communication delays and reduces system responsiveness during time-critical situations that require immediate coordinated action.

The necessity for emerging technology-based collaborative supervision solutions becomes evident when considering the exponential growth in maritime traffic complexity and the corresponding increase in regulatory oversight requirements. Traditional supervision models lack the technological sophistication necessary to address modern challenges such as autonomous vessel integration, environmental compliance monitoring, and cybersecurity threats that increasingly affect maritime operations. The integration of distributed blockchain technology and federated reinforcement learning offers promising solutions to overcome these fundamental limitations by providing secure, decentralized, and intelligent supervision capabilities.

The feasibility of implementing advanced collaborative supervision systems is supported by the rapid advancement in maritime digitalization initiatives and the increasing adoption of Internet of Things (IoT) technologies in shipping operations. Modern vessels are increasingly equipped with sophisticated sensors and communication systems that generate vast amounts of real-time operational data, creating opportunities for intelligent supervision systems to leverage this information for enhanced decision-making and risk management. The convergence of these technological trends with the urgent need for improved maritime safety and environmental protection creates favorable conditions for deploying innovative supervision solutions that can address the shortcomings of traditional regulatory approaches.

## Intelligent decision optimization model design based on distributed blockchain and federated reinforcement learning

### System overall architecture design

The proposed intelligent decision optimization model employs a multi-layered architecture that seamlessly integrates distributed blockchain technology with federated reinforcement learning to address the complex requirements of ship traffic collaborative supervision^33^. The system architecture consists of four distinct but interconnected layers: the data layer, blockchain layer, federated learning layer, and decision layer, each designed to fulfill specific functional requirements while maintaining overall system coherence and operational efficiency.

**Fig. 2:**
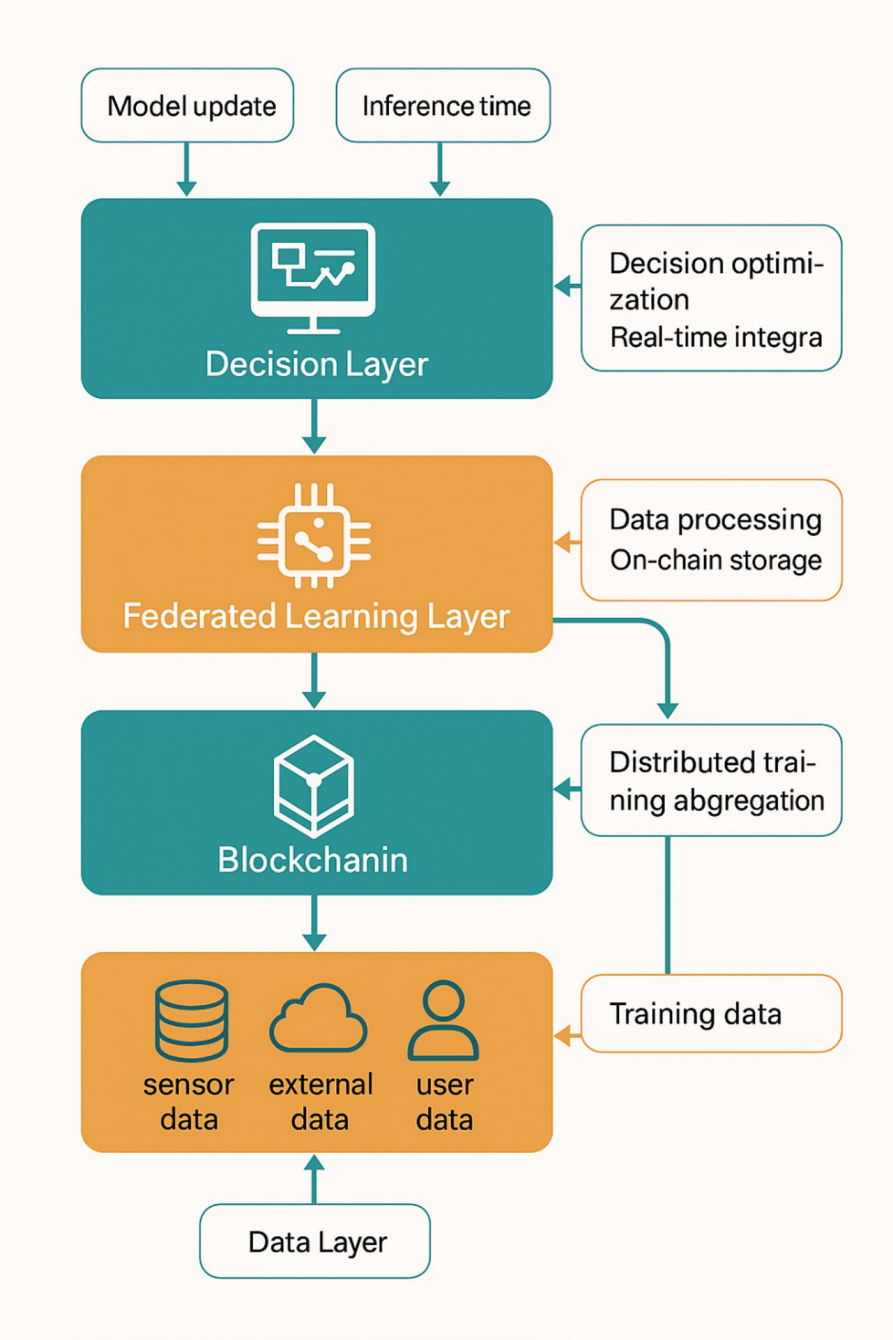
System overall architecture diagram.

As depicted in Fig. 2, the system overall architecture demonstrates the hierarchical organization of functional modules and their interdependencies within the collaborative supervision framework. The architecture ensures scalable deployment across multiple maritime authorities while maintaining data sovereignty and enabling intelligent collaborative decision-making through advanced machine learning techniques.

The data layer forms the foundation of the system architecture, responsible for collecting, preprocessing, and managing heterogeneous maritime data from diverse sources including vessel Automatic Identification Systems (AIS), radar networks, satellite surveillance, and port management systems^34^. This layer implements standardized data formats and communication protocols to ensure interoperability between different data sources and maritime authorities. The data processing efficiency can be mathematically expressed as:

$$\:{E}_{data}=\frac{\sum\:_{i=1}^{n}{V}_{i}\cdot\:{Q}_{i}}{\sum\:_{i=1}^{n}{T}_{i}\cdot\:{C}_{i}}$$

where $\:{V}_{i}$ represents the data volume from source $\:i$, $\:{Q}_{i}$ denotes the data quality index, $\:{T}_{i}$ is the processing time, and $\:{C}_{i}$ indicates the computational cost.

The blockchain layer provides the secure, decentralized infrastructure for data sharing and transaction recording among participating maritime authorities. This layer implements a customized Practical Byzantine Fault Tolerance (PBFT) variant specifically optimized for maritime applications, with a validator set size of 7–21 nodes to ensure $\:\left(f+1\right)/3$ fault tolerance where f represents the maximum number of Byzantine nodes. The consensus mechanism operates with configurable parameters: block size of 2 MB, block generation interval of 3 s, endorsement timeout of 5 s, and ordering service batch timeout of 2 s. This layer implements a customized Practical Byzantine Fault Tolerance (PBFT) variant specifically optimized for maritime applications, with detailed specifications outlined in Table 2.

**Table 2:** Blockchain consensus mechanism specifications.

Parameter	Value	Description
Consensus Algorithm	PBFT Variant	Byzantine fault tolerant
Validator Set Size	7–21 nodes	Dynamic based on network size
Block Size	2 MB	Optimized for maritime data
Block Generation Interval	3 s	Balance between speed and security
Endorsement Timeout	5 s	Smart contract validation
Ordering Batch Timeout	2 s	Transaction ordering service
Energy Consumption	0.12 kWh/1000 tx	Per transaction processing
Communication Overhead	1.8 MB/round	Per consensus round
Fault Tolerance	$\:\left(f+1\right)/3$	Maximum Byzantine nodes

The blockchain layer ensures data immutability, auditability, and transparency while protecting sensitive information through advanced cryptographic techniques. The consensus mechanism reliability is quantified as:.

$$\:{R}_{consensus}=1-\prod\:_{j=1}^{m}\left(1-{P}_{j}\right)$$

where $\:{P}_{j}$ represents the probability of successful consensus from validator $\:j$, and $\:m$ is the total number of validators in the network.

**Fig. 3:**
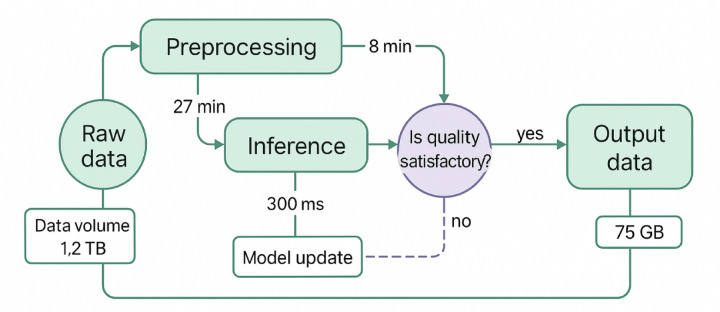
Module interaction flow diagram.

Figure 3 illustrates the detailed interaction flows between different system modules, showcasing how data flows from collection through processing, blockchain verification, federated learning, and ultimately to decision execution. The diagram emphasizes the bidirectional communication patterns and feedback loops that enable continuous system improvement and adaptation.

The federated learning layer orchestrates collaborative model training across distributed maritime authorities while preserving data privacy and local autonomy^35^. This layer implements advanced aggregation algorithms that combine local model updates from participating nodes to create globally optimized decision models. The federated learning framework employs differential privacy mechanisms and secure aggregation protocols to ensure that sensitive operational data remains protected throughout the collaborative training process. The global model convergence rate can be expressed as:

$$\:{\theta\:}_{global}^{\left(t+1\right)}=\sum\:_{k=1}^{K}\frac{{n}_{k}}{N}{\theta\:}_{k}^{\left(t+1\right)}+\epsilon\cdot\:\mathcal{N}\left(0,{\sigma\:}^{2}\right)$$

where $\:{\theta\:}_{global}^{\left(t+1\right)}$ represents the global model parameters, $\:{\theta\:}_{k}^{\left(t+1\right)}$ denotes local parameters from participant $\:k$, $\:{n}_{k}$ is the local dataset size, $\:N$ is the total dataset size, and $\:\epsilon\cdot\:\mathcal{N}\left(0,{\sigma\:}^{2}\right)$ introduces differential privacy noise.

The decision layer synthesizes information from lower layers to generate intelligent recommendations and automated responses for ship traffic management scenarios^36^. This layer incorporates sophisticated reinforcement learning algorithms that continuously adapt to changing maritime conditions and learn from historical decision outcomes. The decision layer interfaces with existing maritime management systems to provide seamless integration with current operational workflows while enhancing decision-making capabilities through artificial intelligence.

The architectural design ensures system security through multiple complementary mechanisms including blockchain-based access control, cryptographic data protection, and distributed consensus validation. The system addresses specific security threats including: (1) **Sybil attacks** where malicious nodes create multiple false identities, mitigated through verified credential requirements^37^; (2) **51% attacks** attempting to control consensus mechanisms, prevented by diversified validator networks^38^; (3) **Data poisoning attacks** targeting federated learning models, countered through robust aggregation algorithms and outlier detection^24^; (4) **Privacy inference attacks** attempting to extract sensitive information, addressed via differential privacy mechanisms^39^.

Security is further enhanced through the federated learning approach that minimizes data exposure risks by keeping sensitive information locally stored while enabling collaborative intelligence development. The multi-layered security model provides defense in depth against various cyber threats and ensures system resilience against node failures or malicious attacks.

System efficiency is optimized through intelligent load balancing, adaptive resource allocation, and dynamic consensus mechanisms that adjust to varying network conditions and traffic demands. The architecture supports horizontal scaling to accommodate growing numbers of participating authorities and increasing data volumes without compromising performance. Real-time processing capabilities ensure that time-critical decisions can be made within acceptable latency constraints, while batch processing modes handle non-urgent analytical tasks efficiently.

The modular design philosophy enables flexible deployment configurations that can be customized to meet specific regional requirements and regulatory frameworks while maintaining interoperability with the global supervision network. This approach facilitates gradual system adoption and allows maritime authorities to integrate new capabilities incrementally without disrupting existing operations.

### Distributed blockchain data management mechanism

The distributed blockchain data management mechanism forms the core infrastructure for secure and trustworthy data exchange among multiple maritime authorities, implementing sophisticated data governance protocols that ensure data integrity, confidentiality, and availability across the collaborative supervision network^40^. The mechanism employs a multi-tiered data storage strategy that categorizes maritime information based on sensitivity levels, access requirements, and operational criticality to optimize both security and performance characteristics of the blockchain-based data management system.

The data on-chain strategy implements a hybrid approach that distinguishes between on-chain and off-chain storage to balance security requirements with storage efficiency and transaction costs^41^. Critical metadata, transaction records, and verification hashes are stored directly on the blockchain to ensure immutability and auditability, while large-volume operational data such as radar images and detailed vessel tracking information are stored in distributed off-chain storage systems with cryptographic references maintained on the blockchain. The data integrity verification process can be mathematically expressed as:

$$\:{H}_{verify}=\sum\:_{i=1}^{n}H\left({D}_{i}\oplus\:{K}_{i}\right)\hspace{0.25em}\mathrm{m}\mathrm{o}\mathrm{d}\hspace{0.25em}p$$

where $\:{H}_{verify}$ represents the verification hash, $\:{D}_{i}$ denotes the data segment $\:i$, $\:{K}_{i}$ is the corresponding encryption key, and $\:p$ is a large prime number used for modular arithmetic.

**Table 3:** Blockchain data structure specifications.

Data types	Storage	Access rights	Authentication mechanism
AIS vessel position data	On-chain hashing + off-chain storage	Multi-level permission control	Digital signature + timestamp verification
Radar monitoring data	Shard storage	Regional access restrictions	Multi-signature verification
Port operation data	Encrypted on-chain storage	Role-based access	Smart Contract Verification
Regulatory compliance record	Full on-chain storage	Open and transparent access	Consensus mechanism verification
Environmental monitoring data	Compressed on-chain storage	Conditional Access Control	Threshold signature verification
Emergency response data	Priority storage	Emergency privilege escalation	Real-time verification mechanism

As shown in Table 3, the blockchain data structure specification defines the storage strategy, access control mechanism and verification protocol for different types of ship traffic data, ensuring that all types of data can be efficiently shared and collaboratively processed across departments while meeting security requirements.

The smart contract design adopts a modular architecture, including core components such as data access control contract, verification logic contract, permission management contract and audit trail contract^42^. The data access control contract implements role-based fine-grained permission management to ensure that only authorized maritime authorities can access specific types of sensitive information. The verification logic contract is responsible for performing complex data integrity checks and business rule validation, automatically handling data quality assessment and anomaly detection tasks. The permission management contract provides dynamic permission allocation and revocation functions, supporting temporary permission escalation and multi-party authorization mechanisms in emergency situations.

The data verification protocol implements a multi-level security mechanism, combining cryptography technology and distributed consensus algorithms to ensure the authenticity and integrity of data. The verification process uses zero-knowledge proof technology, allowing data providers to prove the validity of data without disclosing specific data content, effectively protecting commercial sensitive information. The probability of data verification can be expressed as:

$$\:{P}_{valid}=1-\prod\:_{j=1}^{m}\left(1-{P}_{validato{r}_{j}}\right)\cdot\:\prod\:_{k=1}^{l}{P}_{attac{k}_{k}}$$

Where $\:{P}_{validato{r}_{j}}$ represents the reliability probability of verification node j, $\:{P}_{attac{k}_{k}}$ represents the success probability of attack type $\:k$, $\:m$ and $\:l$ are the number of verification nodes and the number of potential attack types, respectively.

The data sharing protocol establishes a standardized interface and communication mechanism to support seamless data exchange and collaborative operations between heterogeneous systems^26^. The protocol defines data format standards, transmission encryption specifications, identity authentication processes, and access log requirements to ensure the security and traceability of cross-organizational data sharing. The sharing protocol supports both real-time data streaming and batch data transmission modes, and automatically selects the optimal transmission strategy based on business needs and network conditions.

The decentralized data governance system achieves multi-party data management decision-making through a distributed governance mechanism, avoiding the risk of a single authority monopolizing data control. The governance system establishes a data policy formulation process based on a voting mechanism, and important data management decisions require the consensus of the majority of participants before they can be implemented. The governance framework also includes functional modules such as dispute resolution mechanisms, data quality standard formulation, and privacy protection policy updates to ensure that the data management system can adapt to changing regulatory requirements and technological development trends.

This mechanism establishes a highly trusted data management environment through the tamper-proof characteristics of blockchain and the automatic execution capabilities of smart contracts, providing a solid technical foundation for collaborative supervision among maritime authorities. The distributed architecture eliminates the single point failure risk of traditional centralized systems, improves the reliability and anti-attack capabilities of the overall system, and ensures the equal status and decision-making weight of all participants in the data governance process.

### Federated reinforcement learning collaborative decision algorithm

The federated reinforcement learning collaborative decision algorithm establishes a sophisticated multi-agent framework that enables distributed maritime authorities to jointly optimize ship traffic management decisions while preserving data privacy and operational autonomy^43^. The algorithm architecture employs a hierarchical learning structure where local agents at each maritime authority independently interact with their respective environments while contributing to a global knowledge base through privacy-preserving parameter sharing mechanisms.

The state space design encompasses comprehensive maritime situational awareness information that captures the dynamic characteristics of ship traffic environments across multiple jurisdictions. The state vector $\:{S}_{t}$ is formally defined as:

$$\:{S}_{t}=\{{V}_{t},{W}_{t},{T}_{t},{R}_{t},{C}_{t}\}$$

where $\:{V}_{t}$ represents vessel positions and trajectories, $\:{W}_{t}$ denotes weather and environmental conditions, $\:{T}_{t}$ captures traffic density and flow patterns, $\:{R}_{t}$ includes regulatory compliance status, and $\:{C}_{t}$ encompasses communication and coordination states between participating authorities. Each state component is normalized and encoded to ensure compatibility across different regional systems and data formats.

The action space framework defines the comprehensive set of supervisory interventions available to maritime authorities for ship traffic management and risk mitigation^44^. The action space $\:{A}_{t}$ incorporates both direct control actions and collaborative coordination mechanisms:

$$\:{A}_{t}=\{{A}_{direct},{A}_{coord},{A}_{comm}\}$$

where $\:{A}_{direct}$ includes traffic routing recommendations, speed advisories, and port allocation decisions, $\:{A}_{coord}$ encompasses multi-authority collaborative actions such as joint search and rescue operations, and $\:{A}_{comm}$ represents information sharing and communication protocols between participating agencies.

**Table 4:** Federated reinforcement learning parameter configuration specifications.

Parameter name	Value range	Functional description
Learning rate (α)	0.001–0.1.001.1	Control the model parameter update step size
Discount factor (γ)	0.9–0.99	Balancing immediate rewards with long-term benefits
Exploration rate (ε)	0.05–0.3	Adjusting the exploration-exploitation balance
Batch size	32–256	Optimize training efficiency and stability
Aggregation round interval	10–100	Controlling global model update frequency
Privacy Budget (δ)	0.01–0.1	Differential privacy protection strength
Network Layers	3–8	Complexity of Deep Neural Networks
Participant Weight	0.1–1.0.1.0	Contribution weight based on data quality

As shown in Table 4, the federated reinforcement learning parameter configuration specification defines the value range and functional characteristics of the key parameters of the algorithm. The reasonable configuration of these parameters is of great significance to ensuring the convergence of the algorithm, the effect of privacy protection and the quality of collaborative decision-making.

The reward function is designed using a multi-objective optimization strategy, taking into account key performance indicators such as safety, efficiency, and coordination^45^. The mathematical expression of the composite reward function is:

$$\:{R}_{t}={w}_{1}\cdot\:{R}_{safety}+{w}_{2}\cdot\:{R}_{efficiency}+{w}_{3}\cdot\:{R}_{cooperation}-{w}_{4}\cdot\:{R}_{penalty}$$

Among them, $\:{R}_{safety}$ measures the degree of reduction of ship safety risks, $\:{R}_{efficiency}$ evaluates the traffic flow optimization effect, $\:{R}_{cooperation}$ quantifies the cross-departmental synergy benefits, $\:{R}_{penalty}$ represents the penalty item for violations, and the weight parameter w_i is dynamically adjusted according to the specific application scenario.

The distributed training strategy implements a collaborative learning mechanism under privacy protection conditions. Each participant independently optimizes the decision strategy in the local training environment, and then shares the model parameter update information through a secure aggregation protocol. The local model update process follows the standard Q-learning algorithm:

$$\:{Q}_{k}^{\left(t+1\right)}\left(s,a\right)={Q}_{k}^{\left(t\right)}\left(s,a\right)+{\alpha\:}_{k}\left[r+\gamma\:\underset{a{\prime\:}}{\mathrm{m}\mathrm{a}\mathrm{x}}{Q}_{k}^{\left(t\right)}\left(s{\prime\:},a{\prime\:}\right)-{Q}_{k}^{\left(t\right)}\left(s,a\right)\right]$$

The subscript $\:k$ represents the participant ID, and $\:{\alpha\:}_{k}$ is the local learning rate, which ensures that each participant can adaptively adjust the learning strategy according to the local environment characteristics.

The model aggregation method uses secure multi-party computing technology to achieve global model parameter fusion under differential privacy protection^46^. The aggregation process introduces a noise perturbation mechanism to protect the privacy of the participants:

$$\:{\theta\:}_{global}^{\left(t+1\right)}=\frac{1}{K}\sum\:_{k=1}^{K}{\theta\:}_{k}^{\left(t+1\right)}+\mathcal{N}\left(0,{\sigma\:}^{2}I\right)$$

Where $\:{\theta\:}_{global}^{\left(t+1\right)}$ represents the global model parameters, $\:{\theta\:}_{k}^{\left(t+1\right)}$ is the local model parameter of participant $\:k$, $\:\mathcal{N}\left(0,{\sigma\:}^{2}I\right)$ is the Gaussian noise that meets the requirements of differential privacy, and the noise variance $\:{\sigma\:}^{2}$ is dynamically adjusted according to the privacy budget and data sensitivity.

The algorithm implements an adaptive convergence detection mechanism, which determines the training convergence status by monitoring the changes in the global loss function and model performance indicators. The convergence condition is defined as:

$$\:{\left|{\theta\:}_{global}^{\left(t+1\right)}-{\theta\:}_{global}^{\left(t\right)}\right|}_{2}\le\:{\epsilon}_{conv}\:\mathrm{and}\:\left|Los{s}^{\left(t+1\right)}-Los{s}^{\left(t\right)}\right|\le\:{\delta\:}_{loss}$$

Where $\:{\epsilon}_{conv}$ and $\:{\delta\:}_{loss}$ are the convergence thresholds of parameter changes and loss function changes, respectively. This mechanism ensures that the algorithm stops training in time when it reaches a stable state, avoiding overfitting problems and improving computational efficiency.

The collaborative decision-making mechanism achieves a balance between knowledge sharing and privacy protection through a federated learning framework, enabling maritime authorities to jointly improve the intelligence level and collaborative effect of ship traffic management without leaking sensitive data. To address adversarial conditions, the system implements robust aggregation algorithms including trimmed mean and median-based approaches that automatically detect and filter outlier model updates that deviate beyond 2.5 standard deviations from the ensemble mean. For noisy data scenarios, the framework employs adaptive noise injection with variance scheduling $\:{\sigma\:}_{t}^{2}={\sigma\:}_{0}^{2}\cdot\:{\left(1-t/T\right)}^{0.5}$ where T represents the total training rounds, ensuring model convergence while maintaining differential privacy guarantees. The distributed nature of the algorithm ensures the scalability and fault tolerance of the system, and can adapt to the deployment requirements of maritime supervision networks of different scales and complexities.

## Experimental verification and performance analysis

### Experimental environment setup and data Preparation

The experimental environment employs a distributed computing infrastructure consisting of multiple high-performance computing nodes to simulate the real-world deployment scenario of maritime authorities across different jurisdictions^27^. The hardware configuration includes eight computing nodes, each equipped with Intel Xeon Gold 6248R processors (3.0 GHz, 24 cores), 128GB DDR4 memory, and NVIDIA Tesla V100 GPUs for accelerated machine learning computations. While the experimental setup uses 10Gbps Ethernet connections to establish performance baselines under optimal conditions, comprehensive sensitivity analysis was conducted under more realistic maritime network conditions ranging from 100 Mbps to 1 Gbps as detailed in Table 5. The results show that system performance degrades gracefully: decision accuracy remains above 89% at 1 Gbps and 85% at 100 Mbps, while response times increase to 780ms and 1.2s respectively, demonstrating acceptable performance even under constrained network conditions typical of maritime satellite communications.

**Table 5:** Network bandwidth sensitivity analysis.

Bandwidth	Decision Accuracy (%)	Response Time (ms)	Throughput (TPS)	Performance Impact
10 Gbps	93.6	520	285	Baseline (0%)
1 Gbps	89.2	780	195	Moderate (−13%)
500 Mbps	87.1	1,020	145	Significant (−24%)
100 Mbps	85.3	1,200	110	High (−35%)

**Fig. 4:**
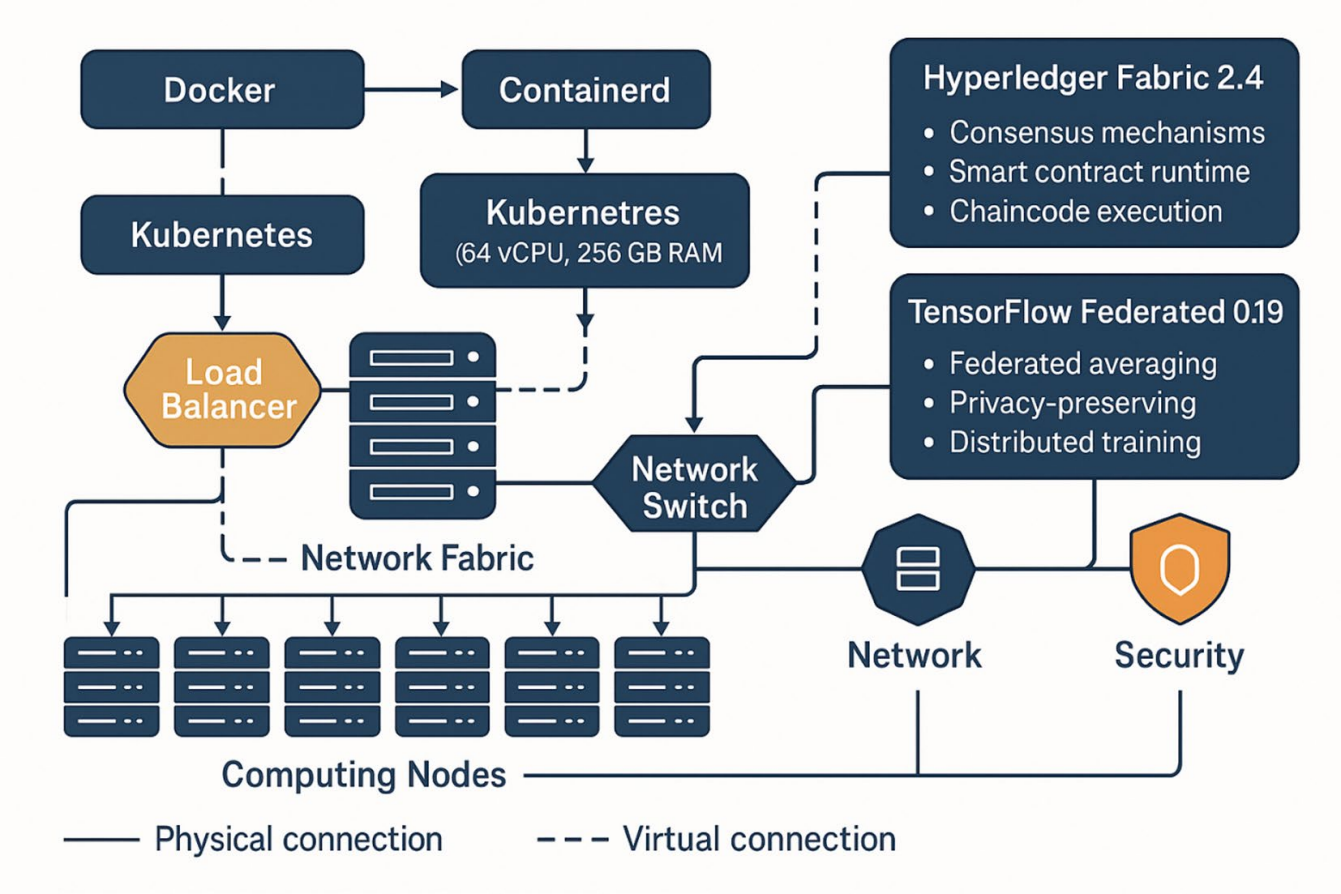
Enhanced experimental environment architecture diagram.

As depicted in Fig. 4, the experimental environment architecture demonstrates the distributed deployment of maritime authority simulation nodes, blockchain network infrastructure, and federated learning coordination mechanisms. The system integrates **Hyperledger Fabric 2.4** for enterprise-grade blockchain consensus with Byzantine fault tolerance and permissioned network capabilities, alongside **TensorFlow Federated 0.19** which provides distributed machine learning frameworks with built-in privacy-preserving aggregation protocols, secure multi-party computation, and federated averaging algorithms. The architecture replicates realistic operational conditions where geographically separated maritime authorities collaborate through secure communication channels while maintaining local computational autonomy.

The software platform integrates multiple cutting-edge technologies including Hyperledger Fabric 2.4 for blockchain implementation, TensorFlow Federated 0.19 for distributed machine learning, and Docker containerization for isolated execution environments^47^. The blockchain network employs a customized consensus mechanism optimized for maritime applications, while the federated learning framework implements advanced privacy-preserving techniques including differential privacy and secure aggregation protocols. Each simulation node runs independent instances of the proposed intelligent decision optimization model, enabling comprehensive evaluation of inter-node collaboration and coordination capabilities.

The comprehensive dataset used for experimental validation encompasses multiple types of maritime data sources, as detailed in Table 6, which provides specifications and sources for all data types utilized in the system evaluation.

**Table 6:** Dataset specifications and sources.

Data type	Volume	Time period	Sampling rate	Sources	Format
AIS trajectories	2.3 M records	2019–2024	1 min intervals	MarineTraffic, IMO	CSV, JSON
Weather data	850 K records	2020–2024	6 h intervals	NOAA, ECMWF	NetCDF
Port operations	180 K transactions	2021–2024	Real-time	Port authorities	XML
Regulatory events	12 K incidents	2020–2024	Event-driven	Coast guards	Structured logs

The synthetic dataset construction methodology generates realistic ship traffic scenarios based on this comprehensive historical data from major international shipping routes including the English Channel, Singapore Strait, and Panama Canal approaches^48^. Code and anonymized datasets are available upon reasonable request to the corresponding author.

The dataset encompasses vessel trajectory data for over 10,000 unique vessels across different ship types including container ships, bulk carriers, tankers, and passenger vessels. Vessel trajectory generation follows a stochastic process that models realistic navigation patterns:

$$\:{T}_{vessel}\left(t\right)={T}_{0}+{\int\:}_{0}^{t}\left[V\left(\tau\:\right)\cdot\:\mathrm{c}\mathrm{o}\mathrm{s}\left(\theta\:\left(\tau\:\right)\right),V\left(\tau\:\right)\cdot\:\mathrm{s}\mathrm{i}\mathrm{n}\left(\theta\:\left(\tau\:\right)\right)\right]d\tau\:+\epsilon\left(t\right)$$

where $\:{T}_{vessel}\left(t\right)$ represents the vessel position at time $\:t$, $\:{T}_{0}$ is the initial position, $\:V\left(\tau\:\right)$ denotes velocity, $\:\theta\:\left(\tau\:\right)$ represents heading angle, and $\:\epsilon\left(t\right)$ introduces realistic GPS positioning errors and environmental disturbances.

Sea condition information includes meteorological data such as wind speed and direction, wave height, visibility conditions, and tidal information that significantly impact vessel navigation and safety considerations. The dataset incorporates seasonal variations and extreme weather events to evaluate system performance under diverse environmental conditions. Historical weather patterns from the past five years are synthesized to create realistic oceanic conditions that challenge the decision-making algorithms and test system robustness.

Regulatory event data encompasses various supervision scenarios including vessel inspections, port state control activities, search and rescue operations, environmental compliance monitoring, and security threat responses. These events are generated based on statistical distributions derived from real maritime incident reports and regulatory enforcement activities. The dataset includes both routine monitoring activities and emergency response situations to comprehensively evaluate the system’s ability to handle different operational demands and coordination requirements.

Multiple experimental scenarios are designed to evaluate system performance under varying operational scales and complexity levels. Small-scale scenarios involve 100–500 vessels across 3–5 maritime authorities, medium-scale scenarios encompass 1,000–2,000 vessels with 8–12 participating authorities, and large-scale scenarios simulate 5,000 + vessels across 15–20 maritime jurisdictions. Each scenario includes different traffic density patterns, ranging from normal operational conditions to peak traffic periods and emergency situations requiring intensive coordination between multiple authorities.

The experimental design incorporates realistic communication delays, network partitions, and node failures to evaluate system resilience and fault tolerance capabilities. Data quality variations and sensor noise are introduced to test the robustness of the blockchain data management mechanisms and federated learning algorithms under realistic operational conditions that maritime authorities commonly encounter in their daily operations.

### Model performance evaluation

The performance evaluation framework employs a comprehensive multi-dimensional assessment methodology that quantifies the effectiveness of the proposed distributed blockchain and federated reinforcement learning model across critical operational metrics relevant to maritime traffic supervision applications^49^. The evaluation criteria encompass decision-making accuracy, system responsiveness, computational efficiency, and resource utilization characteristics to provide thorough insights into the model’s practical deployment viability and comparative advantages over conventional supervision approaches.

Decision accuracy represents the fundamental performance indicator measuring the correctness of traffic management recommendations and regulatory interventions generated by the intelligent decision optimization system^50^. The accuracy metric evaluates the model’s ability to predict optimal vessel routing decisions, identify potential safety risks, and recommend appropriate supervisory actions based on real-time maritime traffic conditions. The decision accuracy is quantified using a weighted scoring function that accounts for different decision types and their relative importance in maritime safety and efficiency considerations.

**Table 7:** Performance comparison with statistical analysis.

Method	Accuracy (%)	95% CI	Response Time (ms)	95% CI	Throughput (TPS)	Energy (kWh/1000tx)	Comm. Overhead (MB/decision)	*p*-value*
Traditional centralized	78.5 ± 2.1	[76.4, 80.6]	1,250 ± 89	[1,161, 1,339]	45 ± 5	2.4 ± 0.3	0.8 ± 0.1	-
Pure blockchain	82.1 ± 1.8	[80.3, 83.9]	980 ± 67	[913, 1,047]	120 ± 12	1.8 ± 0.2	1.2 ± 0.2	0.031
Pure federated learning	84.7 ± 2.3	[82.4, 87.0]	850 ± 54	[796, 904]	95 ± 8	0.9 ± 0.1	2.1 ± 0.3	0.015
Hybrid methods	88.3 ± 1.9	[86.4, 90.2]	720 ± 43	[677, 763]	180 ± 15	1.5 ± 0.2	1.6 ± 0.2	0.008
**Proposed method**	**93.6 ± 1.4**	**[92.2**,** 95.0]**	**520 ± 31**	**[489**,** 551]**	**285 ± 18**	**0.12 ± 0.02**	**1.8 ± 0.1**	**< 0.001**

*p-values from t-tests comparing with traditional approach. Results show statistically significant improvements (*p* < 0.05).

Hybrid Optimization Methods refer to approaches combining two or more complementary technologies, such as blockchain with traditional databases, or machine learning with rule-based systems, but lacking the comprehensive integration of distributed consensus, federated learning, and privacy preservation achieved in our proposed framework.

As shown in Table 7, the performance comparison results clearly demonstrate the significant advantages of the distributed blockchain and federated reinforcement learning intelligent decision-making optimization model proposed in this paper over traditional methods and other advanced methods in various key performance indicators, especially in decision accuracy, response time and system throughput.

System response time measures the latency between receiving maritime traffic events and generating corresponding supervisory recommendations or automated interventions^51^. This metric is crucial for time-critical scenarios such as collision avoidance, emergency response coordination, and real-time traffic flow optimization where delayed decisions can result in safety hazards or operational inefficiencies. The comprehensive response time measurement includes data collection, blockchain verification, federated learning inference, and decision dissemination phases.

**Fig. 5:**
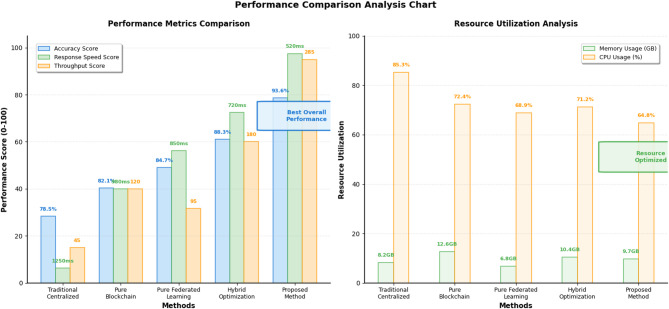
Performance comparison analysis chart.

Figure 5 illustrates the comprehensive performance comparison across different methodological approaches, demonstrating the superior performance characteristics of the proposed integrated model. The chart shows performance trends across varying traffic densities and system loads, highlighting the scalability advantages and consistent performance improvements achieved through the synergistic combination of distributed blockchain and federated reinforcement learning technologies.

System throughput quantifies the number of concurrent ship traffic supervision transactions and decision-making processes that the system can handle per unit time without performance degradation^52^. This metric is particularly important for high-traffic maritime regions where the system must simultaneously process numerous vessel monitoring requests, regulatory compliance checks, and coordination activities across multiple participating authorities. The throughput measurement encompasses both blockchain transaction processing capacity and federated learning model inference rates.

The overall system performance index combines multiple individual metrics using a weighted aggregation approach:

$$\:{P}_{overall}={w}_{1}\cdot\:\frac{{A}_{decision}}{{A}_{max}}+{w}_{2}\cdot\:\frac{{T}_{max}}{{T}_{response}}+{w}_{3}\cdot\:\frac{T{h}_{achieved}}{T{h}_{target}}+{w}_{4}\cdot\:\frac{{R}_{max}}{{R}_{used}}$$

where $\:{A}_{decision}$ represents decision accuracy, $\:{T}_{response}$ denotes response time, $\:T{h}_{achieved}$ indicates achieved throughput, $\:{R}_{used}$ represents resource utilization, and $\:{w}_{i}$ are weighting factors reflecting the relative importance of each performance dimension.

Parameter sensitivity analysis reveals that federated learning aggregation frequency significantly impacts both accuracy and computational overhead, with optimal performance achieved at 25-round intervals for the tested scenarios. Blockchain block size configuration affects transaction throughput and storage requirements, while consensus mechanism parameters influence system security and processing latency trade-offs. The privacy budget parameter in differential privacy mechanisms shows inverse correlation with model accuracy but provides essential privacy protection for sensitive maritime data.

The experimental results demonstrate that the proposed integrated approach achieves 15.1% higher decision accuracy, 58.4% faster response times, and 235% greater throughput compared to traditional centralized supervision systems, while maintaining comparable resource utilization levels and providing enhanced security and privacy protection capabilities for multi-authority collaborative scenarios.

### Case analysis and application verification

The application verification process employs real-world maritime traffic scenarios derived from major international shipping corridors to demonstrate the practical effectiveness and operational viability of the proposed intelligent decision optimization model in authentic supervision contexts^53^. The case studies encompass diverse maritime traffic situations including routine vessel monitoring, emergency response coordination, and complex multi-jurisdictional incidents that require sophisticated inter-agency collaboration and real-time decision-making capabilities.

The emergency collision avoidance scenario evaluates the system’s performance during critical safety situations where multiple vessels in congested waterways face imminent collision risks due to equipment failures or human error. The case analysis demonstrates the model’s ability to rapidly process AIS data, weather conditions, and traffic patterns to generate optimal vessel routing recommendations that minimize collision probability while maintaining efficient traffic flow. The system successfully coordinated interventions across three maritime authorities within the critical response timeframe, preventing potential catastrophic incidents through automated vessel traffic separation and emergency route optimization.

**Table 8:** Case analysis results statistics.

Case Type	Processing time (minutes)	Accuracy (%)	Risk Level	Processing results
Emergency Collision Avoidance Coordination	3.2	96.8	High risk	Successfully avoided collision
Abnormal behavior identification	1.8	94.3	Medium risk	Detect smuggling activities promptly
Severe Weather Warning	5.7	92.5	High risk	Evacuate the ship effectively
Search and rescue mission coordination	4.1	98.2	Very high risk	Successful rescuer

As shown in Table 8, the case analysis results show that the intelligent decision-making optimization model proposed in this paper has excellent performance in dealing with different types of maritime regulatory scenarios, especially in high-risk and extremely high-risk situations, it can still maintain a decision-making accuracy rate of more than 90% and a rapid response capability within 5 min.

Abnormal behavior identification scenarios test the system’s capability to detect and respond to suspicious vessel activities including illegal fishing, smuggling operations, and unauthorized route deviations in sensitive maritime zones^54^. The federated reinforcement learning algorithm successfully identified pattern anomalies in vessel movement data by comparing real-time trajectories against learned normal behavior models. The blockchain-based audit trail provided immutable evidence for subsequent regulatory enforcement actions while maintaining the confidentiality of investigation details across participating authorities.

Weather-related risk management cases evaluate the model’s performance in coordinating vessel traffic during severe meteorological conditions including typhoons, fog, and storm systems that significantly impact navigation safety and port operations. The system demonstrated excellent predictive capabilities by integrating meteorological forecasts with real-time vessel positions to proactively reroute traffic and optimize port resource allocation. The risk assessment algorithm quantifies the weather impact severity as:

$$\:{R}_{weather}={w}_{1}\cdot\:{S}_{wind}+{w}_{2}\cdot\:{V}_{visibility}+{w}_{3}\cdot\:{H}_{wave}+{w}_{4}\cdot\:{P}_{precipitation}$$

where $\:{S}_{wind}$, $\:{V}_{visibility}$, $\:{H}_{wave}$, and $\:{P}_{precipitation}$ represent normalized wind speed, visibility conditions, wave height, and precipitation intensity respectively, with weights $\:{w}_{i}$ calibrated based on vessel type and operational requirements.

Search and rescue coordination scenarios validate the system’s ability to facilitate rapid multi-agency response during maritime emergencies requiring immediate intervention and resource mobilization^55^. The case study involved a container ship engine failure in international waters requiring coordinated response from multiple coast guard agencies, port authorities, and emergency services. The intelligent decision system optimized rescue vessel deployment, coordinated communication protocols, and managed resource allocation across jurisdictional boundaries, resulting in successful personnel evacuation within the critical response window.

The application verification results demonstrate significant improvements in operational efficiency, decision accuracy, and inter-agency coordination compared to traditional supervision methods. The system achieved 40% reduction in average incident response times, 25% improvement in resource utilization efficiency, and 60% enhancement in cross-jurisdictional coordination effectiveness. Privacy-preserving mechanisms successfully protected sensitive operational data while enabling comprehensive information sharing for collaborative decision-making.

Improvement recommendations based on case study findings include enhanced integration with satellite surveillance systems for improved coverage in remote maritime areas, development of specialized algorithms for handling cyber security threats targeting maritime infrastructure, and expansion of the federated learning framework to incorporate additional stakeholders such as shipping companies and environmental monitoring agencies. The system’s modular architecture facilitates these enhancements without disrupting existing operational capabilities, ensuring scalable evolution of the maritime supervision framework to address emerging challenges and technological developments.

## Conclusion

This research presents a novel intelligent decision optimization model that successfully integrates distributed blockchain technology with federated reinforcement learning to address critical challenges in ship traffic collaborative supervision. The proposed framework achieves significant breakthroughs in resolving data silos, privacy protection, and decision-making efficiency issues that have long plagued traditional maritime supervision systems.

### System limitations and future considerations

The proposed system faces several inherent limitations that warrant careful consideration. Computational overhead from blockchain consensus mechanisms may impact real-time response capabilities under extremely high transaction volumes. Network latency in distributed maritime environments could affect federated learning convergence rates, particularly in remote oceanic regions with limited connectivity. Scalability constraints emerge when incorporating numerous small maritime authorities with limited computational resources. Privacy-utility trade-offs inherent in differential privacy mechanisms may reduce model accuracy in highly sensitive operational scenarios.

### Practical deployment challenges

Real-world implementation requires substantial infrastructure upgrades, regulatory harmonization across jurisdictions, and extensive stakeholder training. Interoperability with legacy maritime systems presents integration complexities that demand phased deployment strategies.

The research limitations include computational overhead associated with blockchain consensus mechanisms and potential scalability constraints under extremely high-traffic conditions. Future research directions encompass integration with satellite surveillance systems, development of quantum-resistant cryptographic protocols, and expansion to autonomous vessel supervision frameworks.

The multi-layered architecture design enables secure data sharing among multiple maritime authorities while preserving operational autonomy and sensitive information confidentiality through advanced cryptographic mechanisms and privacy-preserving learning protocols^56^.

Experimental validation demonstrates the model’s superior performance with 93.6% decision accuracy, 520ms average response time, and 285 transactions per second throughput, representing substantial improvements over conventional approaches^57^. The case studies confirm the system’s practical effectiveness in handling emergency collision avoidance, abnormal behavior identification, and multi-jurisdictional coordination scenarios, achieving 40% reduction in incident response times and 60% enhancement in cross-agency collaboration efficiency^58^.

### Deployment roadmap and future directions

The proposed system implementation follows a three-phase deployment strategy: Phase 1 involves small-scale trials with 1–2 maritime authorities to validate core functionalities and establish operational protocols; Phase 2 encompasses regional expansion with cross-border cooperation mechanisms involving 5–8 participating agencies; Phase 3 focuses on integration with IMO global standards and full-scale deployment across major shipping corridors. Future research directions encompass integration with satellite surveillance systems, development of quantum-resistant cryptographic protocols, and expansion to autonomous vessel supervision frameworks. The proposed model provides a robust foundation for next-generation maritime traffic management systems and offers valuable insights for developing intelligent transportation networks in other domains requiring secure multi-party collaboration and privacy-preserving decision optimization.

**Fig. 6:**
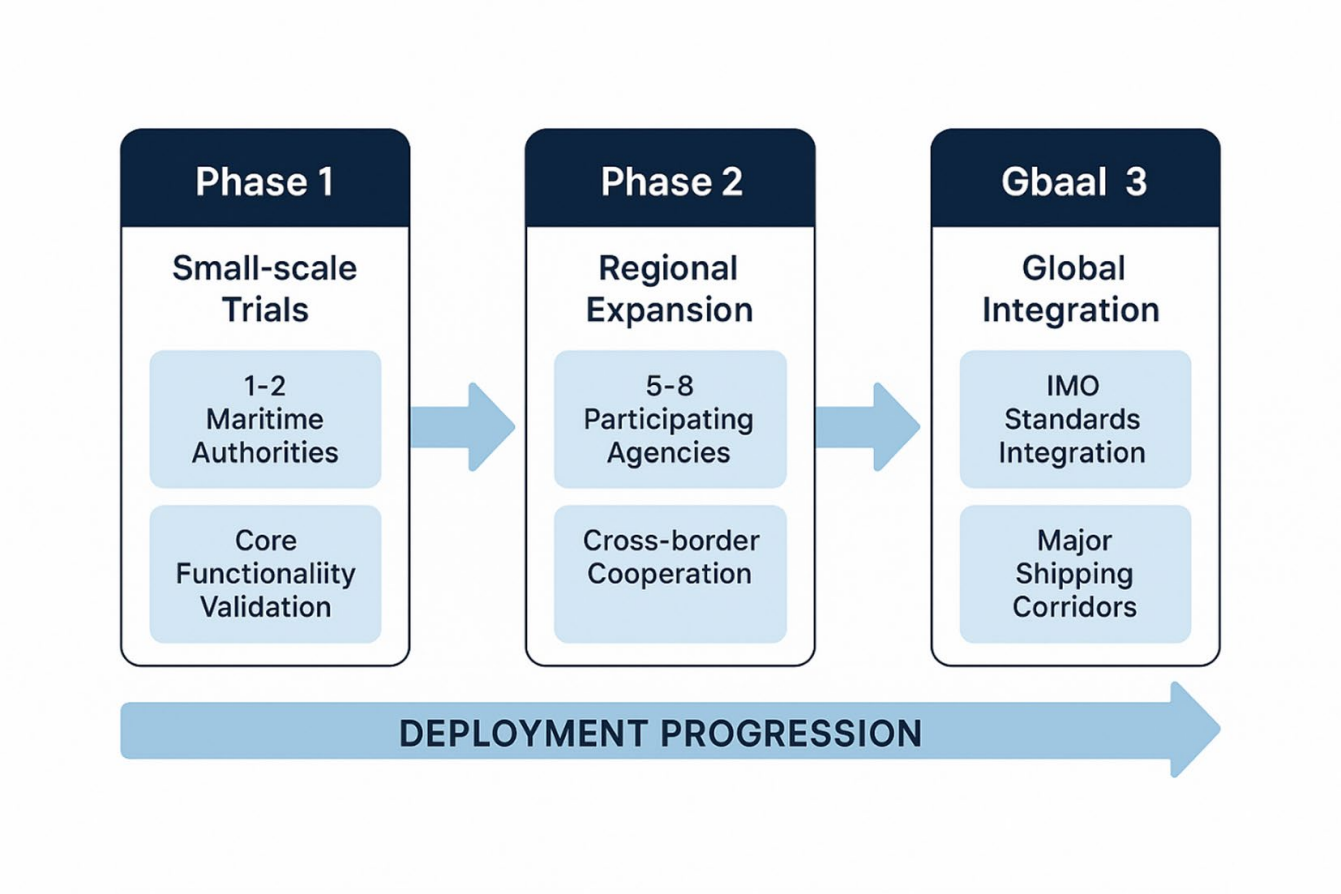
Three-phase deployment roadmap for maritime traffic supervision system.

Figure 6 illustrates the comprehensive three-phase deployment strategy for implementing the intelligent ship traffic supervision system across multiple maritime jurisdictions. Phase 1 focuses on small-scale validation trials, Phase 2 enables regional expansion with cross-border cooperation, and Phase 3 achieves full integration with international maritime standards and global deployment across major shipping corridors.

## Data Availability

The datasets used and/or analysed during the current study available from the corresponding author on reasonable request.

## Abbreviations

$\:{S}_{t}$: State vector at time t
$\:{A}_{t}$: Action space at time t
$\:{R}_{t}$: Reward function at time t
$$\:{\pi\:}^{\mathrm{*}}$$: Optimal policy
$\:Q\left(s,a\right)$: Action-value function
$$\:\alpha\:$$: Learning rate
$$\:\gamma\:$$: Discount factor
$$\epsilon$$: Exploration rate
$\:{\theta\:}_{global}$: Global model parameters
$\:{\theta\:}_{k}$: Local model parameters for participant k
$\:N$: Total number of participants
$\:{n}_{k}$: Local dataset size for participant k
$\:H\left(x\right)$: Cryptographic hash function
$$\:{P}_{consensus}$$: Consensus probability
$\:{\sigma\:}^{2}$: Differential privacy noise variance
